# Drug-drug interactions in atrial fibrillation patients receiving direct oral anticoagulants

**DOI:** 10.1038/s41598-021-01786-2

**Published:** 2021-11-17

**Authors:** Ji Yun Lee, Il-Young Oh, Ju-Hyeon Lee, Seok Kim, Jihoon Cho, Charg Hyun Park, Sooyoung Yoo, Soo-Mee Bang

**Affiliations:** 1 Department of Internal Medicine, Seoul National University Bundang Hospital, Gumi-ro 173 Beon-gil, Bundang-gu, Seongnam-Si, 13620 Gyeonggi-di Republic of Korea; 2 Office of eHealth Research and Business, Seoul National University Bundang Hospital, Seongnam, Republic of Korea

**Keywords:** Medical research, Outcomes research

## Abstract

Polypharmacy is common in patients with atrial fibrillation (AF), making these patients vulnerable to the occurrence of potential drug-drug interactions (DDIs). We assessed the risk of ischemic stroke and major bleeding in the context of concomitant treatment with potential DDIs in patients with AF prescribed direct oral anticoagulants (DOACs). Using the common data model (CDM) based on an electronic health record (EHR) database, we included new users of DOACs from among patients treated for AF between January 2014 and December 2017 (n = 1938). The median age was 72 years, and 61.8% of the patients were males, with 28.2% of the patients having a CHA_2_DS_2_-VASc score in category 0–1, 49.4% in category 2–3, and 22.4% in category ≥ 4. The CHA_2_DS_2_-VASc score was significantly associated with ischemic stroke occurrence and hospitalization for major bleeding. Multiple logistic regression analysis showed that increased risk of ischemic stroke and hospitalization for major bleeding was associated with the number of DDIs regardless of comorbidities: ≥ 2 DDIs was associated with ischemic stroke (OR = 18.68; 95% CI, 6.22–55.27, *P* < 0.001) and hospitalization for major bleeding (OR = 5.01; 95% CI, 1.11–16.62, *P* < 0.001). DDIs can cause reduced antithrombotic efficacy or increased risk of bleeding in AF patients prescribed DOACs.

## Introduction

Atrial fibrillation (AF) is a major public health burden worldwide, and the prevalence of AF is remarkably increasing according to population aging^1,2^. AF increases the risk of ischemic stroke by nearly fivefold and accounts for up to 15% of strokes in people of all ages and 30% in people over the age of 80 years^3,4^. Direct oral anticoagulants (DOACs), including dabigatran, rivaroxaban, apixaban, and edoxaban, are being increasingly prescribed in clinical practice as the preferred class of oral anticoagulants for stroke prevention in non-valvular AF^5–9^. Although DOACs represent an advance in therapeutic safety when compared to warfarin for the prevention of stroke, the appropriateness and accuracy of prescribing medications are important^10,11^.

Drug–drug interactions (DDIs) are a concern for both patients and providers, as polypharmacy is becoming more common in managing complex diseases or comorbidities^12^. Among AF patients included in recent clinical trials, the prevalence of polypharmacy ranged between 40 and 75% and was linked to increased rates of cardiovascular mortality, bleeding, and thromboembolic complications^13–15^. Recently, data on DDIs with DOACs and increased risk of bleeding have emerged from large claims database studies^16,17^. Momo et al. showed that both the pharmacokinetics and the pharmacodynamics of DDIs increased the risk of bleeding in AF patients receiving anticoagulants by about 7-fold^16^. Our group reported an approximately fourfold increase in risk for major bleeding events in DOAC users concomitantly taking ≥ 2 potentially interacting drugs, regardless of comorbidities^17^.

The Observational Health Data Sciences and Informatics (OHDSI) is an international collaborative organization whose goal is to create and apply open-source data analytic solutions to a large network of health databases^18^. OHDSI adopts a distributed research network with the Observational Medical Outcomes Partnership Common Data Model (OMOP-CDM), which allows for the systematic analysis of disparate observational databases for clinical research^19^. Recent studies demonstrated that the use of OMOP-CDM was feasible for pharmacoepidemiologic and pharmacovigilance research^20,21^.

The purpose of this study was to assess whether potential DDIs affect the safety and efficacy of DOACs in patients with AF using a CDM at a single institution.

## Methods

### Data sources

Electronic health records (EHRs) data from Seoul National University Bundang Hospital were transformed into OMOP-CDM version 5.2. Diagnoses were coded according to the 6th Korean Classification of Disease modified classification systems from the International Classification of Disease-10 (ICD-10). Drug names were mapped to the Anatomical Therapeutic Chemical Classification System.

This study was performed in accordance with the Declaration of Helsinki and approved by the Institutional Review Board of Seoul National University Bundang Hospital. The need for informed consent from each patient was waived because the authors did not have access to identifiable information (IRB No: X-19040/535-901).

### Study population

We included patients with diagnostic codes for AF from January 2013 to December 2017 and who received DOAC (rivaroxaban, apixaban, dabigatran, or edoxaban) treatment for 7 days or longer from initial diagnosis (n = 3681). We used several exclusion criteria to maximize data accuracy. First, we excluded AF patients previously prescribed DOAC between January 2013 and December 2013 to analyze only new cases (n = 1188). Second, patients < 20 years of age diagnosed with AF and patients with valvular AF were excluded (n = 2). Third, patients with an alternative indication for DOAC treatment and prophylaxis, including deep vein thrombosis, pulmonary embolism, and joint replacement surgery, were excluded (n = 25). Fourth, patients with end-stage renal disease were excluded (n = 0). Lastly, patients with ischemic stroke (IS), intracranial hemorrhage (ICH), or gastrointestinal (GI) bleeding in the 6 months prior to cohort entry were excluded in the analysis of primary prevention (n = 528). Finally, a total of 1938 patients (diagnosed between January 2014 and December 2017) were selected for this study. The detailed patient enrollment flow is described in Supplementary Figure 1.

### DDIs

Forty-five concurrent medications that may have a potential DDI with DOACs were selected as follows: (1) drugs affecting platelet function such as antiplatelet agents, non-steroidal anti-inflammatory drugs (NSAIDs), and serotonergic agents such as selective serotonin reuptake inhibitors (SSRIs), and serotonin and norepinephrine reuptake inhibitors (SNRIs); (2) P-glycoprotein inhibitors or CYP3A4 inhibitors such as amiodarone, clarithromycin, cobicistat, fluconazole, itraconazole, and voriconazole; and (3) P-glycoprotein inducers or CYP3A4 inducers such as carbamazepine, phenobartibal, phenytoin, and rifampin^22–25^ (Supplementary Table 1).

### Study outcomes

We identified four clinical outcomes as follows: IS, ICH, hospitalization for GI bleeding, and hospitalization for major bleeding. To assess the outcomes, we followed up the patients for 1 year. Detailed definitions of the clinical outcomes are described in Supplementary Table 2.

DOACs administered within 30 days before the clinical outcomes were examined to assess clinical outcomes associated with DOACs. If DOACs were not administered within 30 days before the events, the event was not counted in our analysis. To identify potential drug interactions causing an increased risk of DOAC-related bleeding or a reduced antithrombotic efficacy, we examined the use of DDI drugs 30 days prior to the events.

### Comorbidities

Comorbidities were included in the model as CHA_2_DS_2_-VASc scores by assigning 1 point for age between 65 and 74 years, female sex, hypertension, diabetes, congestive heart failure, or vascular disease, and adding 2 points for age 75 years or older, history of stroke, transient ischemic attack, or systemic thromboembolism^26^. CHA_2_DS_2_-VASc scores were divided into three categories: 0–1, 2–3, and ≥ 4.

### Statistical methods

Patient characteristics were summarized using descriptive statistics. The median and interquartile range (IQR) was reported for continuous variables, and categorical variables were expressed as frequencies (percentage). Multiple logistic regression analysis was performed using a forced entry method to examine the associations of the CHA_2_DS_2_-VASc score and DDIs with the risk for poor clinical outcomes. For each independent variable, the odds ratio (OR) and 95% confidence interval (CI) were determined. All tests were 2-tailed, with *P* < 0.05 considered significant. All statistical analysis was performed using R Statistical Software version 3.6.3 (Vienna, Austria; http://www.R-project.org/).

## Results

### Baseline characteristics

Between 2014 and 2017, a total of 1,938 patients with AF who were newly administered DOACs were included in the study. The clinical baseline characteristics of the study are shown in Table 1. The median age was 72 years, and 61.8% of the patients were males. Among the DOACs, rivaroxaban was the most used (29.4%), followed by apixaban (22.3%) and dabigatran (15.2%). The proportion of subjects in each CHA_2_DS_2_-VASc score category was as follows: 28.2% in category 0–1, 49.4% in category 2–3, and 22.4% in category ≥ 4.

**Table 1:** Baseline characteristics of the study population.

	N	%
**Age, years**		
Median (IQR)	72 (62–78)	
< 65	571	29.5
65–74	621	32.0
≥ 75	746	38.5
**Sex**		
Male	1,198	61.8
Female	740	38.2
**DOAC**		
Rivaroxaban	569	29.4
Apixaban	433	22.3
Dabigatran	295	15.2
Edoxaban	159	8.2
Mixed*	482	24.9
**CHA**_**2**_**DS**_**2**_-**VASc score**		
0–1	546	28.2
2–3	957	49.4
≥ 4	435	22.4

IQR, interquartile range; DOAC, direct oral anticoagulant.

CHA_2_DS_2_-VASc scores indicate congestive heart failure, hypertension, age ≥ 75 years (doubled), diabetes mellitus, prior stroke or transient ischemic attack (doubled), vascular disease, age 65 to 74 years, and female sex.

*Mixed signifies the patient switched DOACs.

### Ischemic stroke

IS events associated with DOACs occurred in 29 patients (1.5%) during the observation period (Table 2). Although not statistically significant, the median age was higher in the IS group than in the group without IS (76 vs. 71 years, *P* = 0.062). The CHA_2_DS_2_-VASc score was significantly associated with IS occurrence; as the score increased, so did the risk of IS. In addition, IS events were more common in patients who simultaneously took drugs with potential DDIs and DOACs, with risk increasing alongside the number of DDIs: 1 (OR = 6.22; 95% CI, 2.65–15.67, *P* < 0.0001) and ≥ 2 (OR = 12.22; 95% CI, 4.21–34.72, *P* < 0.001). The use of P-glycoprotein inducers or CYP3A4 inducers was not observed in patients with IS (Supplementary Table 3). Most of the potential drugs used in patients with IS were identified as pharmacodynamic drugs: antiplatelet agents such as aspirin (n = 15) and clopoidogrel (n = 5) (Supplementary Table 3).

**Table 2:** Comparisons between patients with and without ischemic stroke.

	Ischemic stroke (N = 29)		No ischemic stroke (N = 1,909)		Odds ratio (95% CI)	*P*-value
	N	%	N	%		
Age, median (IQR)	76 (71–80)		71 (62–78)		1.04 (1.00–1.08)	0.062
**Sex**						
Male	18	62.1	1,180	61.8	REF	
Female	11	37.9	729	38.2	0.99 (0.45–2.08)	0.977
**CHA**_**2**_**DS**_**2**_-**VASc **						
0–1	4	13.8	542	28.4	REF	
2–3	10	34.5	947	49.6	1.43 (0.48–5.24)	0.546
≥ 4	15	51.7	420	22.0	4.84 (1.74–17.07)	0.005
**DOAC**						
Rivaroxaban	9	31.0	560	29.3	REF	
Apixaban	1	3.5	432	22.6	0.14 (0.01–0.77)	0.067
Dabigatran	1	3.5	294	15.4	0.21 (0.11–1.13)	0.142
Edoxaban	1	3.5	158	8.3	0.39 (0.02–2.12)	0.378
Mixed*	17	58.6	465	24.4	2.28 (1.03–5.36)	0.049
**DDI**						
No	8	27.6	1,411	73.9	REF	
Yes	21	72.4	498	26.1	7.44 (3.40–17.97)	< 0.001
**Number of DDIs**						
0	8	27.6	1,411	73.9	REF	
1	14	48.3	397	20.8	6.22 (2.65–15.67)	< 0.001
≥ 2	7	24.1	101	5.3	12.22 (4.21–34.72)	< 0.001

IQR, interquartile range; DOAC, direct oral anticoagulant; DDI, drug-drug interaction; REF, reference.

CHA_2_DS_2_-VASc score indicates congestive heart failure, hypertension, age ≥ 75 years (doubled), diabetes mellitus, prior stroke or transient ischemic attack (doubled), vascular disease, age 65 to 74 years, and female sex.

*Mixed signifies the patient switched DOACs.

### Hospitalization for major bleeding

Hospitalization for major bleeding associated with the use of DOACs occurred in 22 patients (1.1%) during the observation period (Table 3). The median age was significantly higher in the hospitalization for major bleeding group than in the group without hospitalization for major bleeding (81 vs. 71 years, *P* < 0.001). When analyzed against the CHA_2_DS_2_-VASc score, events of hospitalization for major bleeding were significantly more likely patients with 4 points or more compared to patients with 0–1 points (OR = 11.51; 95% CI, 2.15–212.75, *P* = 0.021). There was no association between DOAC type and hospitalization for major bleeding events. Hospitalization for major bleeding events had a statistically significant relationship with potential DDIs (OR = 2.81; 95% CI, 1.20–6.60, *P* = 0.016), and the risk tended to increase as the number of DDIs increased. Most of the potential drugs used in patients with hospitalization for major bleeding were antiplatelet agents (n = 4) or NSAIDs (n = 3) (Supplementary Table 4). The relationship between ICH and hospitalization for GI bleeding and DDI is described in Supplementary Tables 5 and 6, respectively. GI bleeding showed a statistically significant relationship with DDI, whereas ICH did not.

**Table 3:** Comparisons between patients with and without hospitalization for major bleeding.

	Hospitalization for major bleeding (N = 22)		No hospitalization for major bleeding (N = 1,916)		Odds ratio (95%CI)	*P*-value
	N	%	N	%		
Age, median (IQR)	81 (75–84)		71 (62–78)		1.13 (1.07–1.19)	< 0.001
**Sex**						
Male	13	59.1	1,185	61.9	REF	
Female	9	40.9	731	38.1	1.12 (0.46–2.61)	0.791
**CHA**_**2**_**DS**_**2**_-**VASc **						
0–1	1	4.6	545	28.4	REF	
2–3	12	54.5	945	49.3	6.92 (1.36–126.26)	0.063
≥ 4	9	40.9	426	22.2	11.51 (2.15–212.75)	0.021
**DOAC**						
Rivaroxaban	5	22.7	564	29.4	REF	
Apixaban	7	31.8	426	22.2	1.85 (0.59–6.30)	0.295
Dabigatran	1	4.6	294	15.3	0.38 (0.02–2.39)	0.383
Edoxaban	0	0	159	8.3	0	0.986
Mixed*	9	40.9	473	24.7	2.15 (0.74–7.03)	0.174
**DDI**						
No	11	50.0	1,413	73.8	REF	
Yes	11	50.0	503	26.2	2.81 (1.20–6.60)	0.016
**Number of DDIs**						
0	11	50.0	1,413	73.7	REF	
1	8	36.4	396	20.7	2.60 (1.00–6.46)	0.042
≥ 2	3	13.6	107	5.6	3.60 (0.81–11.7)	0.052

IQR, interquartile range; DOAC, direct oral anticoagulant; DDI, drug-drug interaction; REF, reference.

CHA_2_DS_2_-VASc indicates congestive heart failure, hypertension, age ≥ 75 years (doubled), diabetes mellitus, prior stroke or transient ischemic attack (doubled), vascular disease, age 65 to 74 years, and female sex.

*Mixed signifies the patient switched DOACs.

### Multiple logistic regression analysis for clinical outcomes

Multiple logistic regression analysis showed that increased risk of IS and hospitalization for major bleeding was associated with the number of DDIs regardless of comorbidities: ≥ 2 DDIs was associated with IS (OR = 18.68; 95% CI, 6.22–55.27, *P* < 0.001) and hospitalization for major bleeding (OR = 5.01; 95% CI, 1.11–16.62, *P* < 0.001) (Table 4).

**Table 4:** Multiple logistic regression analysis for clinical outcomes.

Variables	Ischemic stroke		Hospitalization for major bleeding	
	Odds ratio (95% CI)	*P* value	Odds ratio (95% CI)	*P* value
**CHA**_**2**_**DS**_**2**_-**VASc **				
0–1	REF		REF	
2–3	2.35 (0.77–8.71)	0.157	9.42(1.82–172.79)	0.032
≥ 4	8.27 (2.87–30.03)	< 0.001	15.09 (2.78–280.42)	0.011
**Number of DDIs**				
0	REF		REF	
1	6.92 (2.91–17.61)	< 0.001	3.27 (1.25–8.21)	0.012
≥ 2	18.68 (6.22–55.27)	< 0.001	5.01 (1.11–16.62)	0.016

DDI, drug-drug interaction; REF, reference.

CHA_2_DS_2_-VASc indicates congestive heart failure, hypertension, age ≥ 75 years (doubled), diabetes mellitus, prior stroke or transient ischemic attack (doubled), vascular disease, age 65 to 74 years, and female sex.

## Discussion

Management of oral anticoagulant drug interaction is essential to ensure safe and effective use. Warfarin has over 200 identified drug interactions that must be considered before use^27^. Wang et al. conducted meta-analysis based on low- to moderate-strength evidence supporting interaction between warfarin and a small group of medications leading to bleeding risk or thromboembolic outcomes^28^. Although DOACs have comparable efficacy and enhanced safety compared to warfarin, the appropriateness and accuracy of prescribing medications are important to prevent increased risk of bleeding or reduced antithrombotic efficacy. In the current study, we found that potential DDIs were associated with a substantially high risk for both ischemic stroke and hospitalization for major bleeding regardless of comorbidities.

Drug interactions have been previously associated with decreased potency of DOACs^25^. Based on pharmacokinetic data and published case reports, there is a significant decrease in DOAC drug concentration and an increased risk of adverse thrombotic events in patients receiving concomitant P-glycoprotein inducers or CYP3A4 inducers^29–32^. DDIs were associated with a significantly higher risk for IS, in particular for DDIs with ≥ 2 prescribed drugs (OR, 18.68; 95% CI, 6.22–55.27). Most of the DDIs related to IS were pharmacodynamic drugs such as antiplatelet agents or NSAIDs and were not related to P-glycoprotein inducers or CYP3A4 inducers. The ARISTOTLE trial reported that participants on aspirin were at higher risk for ischemic events, with higher CHADS_2_ scores, than were participants not receiving aspirin^33^. In the current study, concomitant use of antiplatelet agents was observed in patients with IS, which could explain the higher CHA_2_DS_2_-VASc scores of those on antiplatelet agents. Previous studies demonstrated a thrombotic risk associated with NSAIDs^34–36^. Kent et al. demonstrated that the use of NSAIDs was associated with an increased risk for ischemic stroke as well as major bleeding^37^. It is difficult to assess the clinical relevance of DOAC drug interactions because the available data are frequently limited to pharmacokinetic studies in a small number of healthy volunteers or retrospective case–control or cohort studies.

Romoli et al. showed that switching between DOACs is frequent, occurring in up to 11% of patients prescribed with DOAC for AF. In this study, one in 4 patients had to switch between DOACs within 30 days before ischemic stroke or bleeding events. Due to the anonymization of CDM data, the cause of switching between DOACs was not known, but other studies reported that minor bleeding and non-CV adverse events had been reported as one of the most common causes to justify switching between DOACs^38,39^. IS was more common in those who changed DOACs compared to those who took rivaroxaban alone, with an OR of 2.28. Considering that continuous adherence to DOACs is essential to maintain stroke risk reduction^40,41^, the inability to take continuous doses could result in IS events.

In the current study, among those hospitalized for major bleeding, GI bleeding showed a statistically significant relationship with DDIs, but ICH did not. Our group reported that the rate of exposure to DDIs in bleeding events was about 57%, and NSAIDs and antiplatelet agents were the most common drugs for DDIs^17^. Although the data is not presented in this study, NSAIDs and antiplatelet agents caused potential DDIs as their use alongside DOACs was concomitant when GI bleeding occurred. The combination of anticoagulation medication and NSAIDs or aspirin increased the risk of GI bleeding^42,43^. The mechanism for the increased GI bleeding associated with NSAIDs relies on the effect of NSAIDs on platelet aggregation and gastric mucosa^44,45^. In addition, considering the potential renal effect of NSAIDs, it is possible that the exposure increased in the concomitant with DOACs, which are mainly excreted by the kidney^46^.

Our study has several limitations. First, due to the nature of the CDM data, we could not review the medical records of any individual patient such as lifestyle risk factors like cigarette smoking and alcohol consumption or change of DOACs due to serious side effects. Second, according to the recent domestic claim data, less than 10% of AF patients using DOAC had CHA_2_DS_2_-VASc scores of 0–1 ^17,47^, but the current CDM study showed that proportion to be close to 28%. The reason for the higher proportion of patients with CHA2DS2-VASc scores of 0–1 compared to other studies is that the diagnosis of comorbidities such as hypertension and diabetes are likely to be missed. To correct this error, even if there was no diagnosis, CHA2DS2-VASc scores reflected whether a patient was taking medication for hypertension or diabetes. Third, the CDM database did not have data on adherence to prescribed medications and information on other hospital medications. In Korea, there is a Korean Health Insurance Review and Assessment Service database to check the history of drug administration while visiting a hospital for the last 90 days. In the future, the limitations of the CDM database can be overcome by reflecting this information. Fourth, as with all non-experimental studies, associations can be drawn, but causality can only be inferred. The number of DDIs can either decrease or enhance the effect of the involved drugs, increasing the risk of side-effects, as well as indicate the presence of multimorbidity. Further studies are required to better understand DDIs leading to adverse events in patients with polypharmacy. Lastly, the potential DDIs of this study may contain inaccuracies or contradict other interaction databases. In light of these limitations, it is essential to regularly reassess the literature for optimal clinical decision-making.

In conclusion, physicians prescribing DOACs for AF should be aware that DDIs can cause significantly increased risk for both IS and hospitalization for major bleeding regardless of comorbidities.

## Supplementary Information

Supplementary Information.
