# Supplementary material for: Drug-drug interactions in atrial fibrillation patients receiving direct oral anticoagulants
Source: Sci Rep. 2021 Nov 17;11:22403. doi: 10.1038/s41598-021-01786-2 (PMC8599657; doi:10.1038/s41598-021-01786-2)

**ORIGINAL ARTICLE**

**Drug-drug interactions in atrial fibrillation patients receiving direct oral anticoagulants**

Ji Yun Lee^1^, Il-Young Oh^1^, Ju-Hyeon Lee^1^, Seok Kim^2^, Jihoon Cho^2^, ChargHyun Park^2^, Sooyoung Yoo^2^, Soo-Mee Bang^1^

^1^Department of Internal Medicine, Seoul National University, Seoul National University Bundang Hospital, Seongnam, Republic of Korea

^2^Office of eHealth Research and Business, Seoul National University Bundang Hospital, Seongnam, Republic of Korea

**Running title:** Drug-drug interactions with direct oral anticoagulants

^†^**Address for correspondence:** Soo-Mee Bang, MD, PhD

Department of Internal Medicine, Seoul National University Bundang Hospital, Gumi-ro 173 Beon-gil, Bundang-gu, Seongnam-Si, Gyeonggi-di 13620, Korea

Tel: +82-31-787-7039; Fax: +82-31-787-4098

E-mail: smbang7@snu.ac.kr

**Supplement Table 1. Candidate drugs lists for drug-drug interaction (DDI)**

| Pharmacodynamic DDI | |
| --- | --- |
| Antiplatelet agent | Aspirin |
|  | Anagrelide |
|  | Ticlopidine |
|  | Iloprost |
|  | Tirofiban |
|  | Clopidogrel |
|  | Treprostnil |
|  | Prasugrel |
| Non-steroidal anti-inflammatory drugs (NSAIDs) | Naproxen |
|  | Ibuprofen |
|  | Indomethacin |
|  | Ketoprofen |
|  | Ketorolac |
|  | Nabumetone |
|  | Aceclofenac |
|  | Meloxicam |
|  | Morniflumate |
|  | Celecoxib |
|  | Etoricoxib |
| Selective serotonin reuptake inhibitors (SSRIs) | Fluxoxamine |
|  | Fluoxetine |
|  | Paroxetin |
|  | Sertraline |
|  | Escitalopram |
| Serotonin-norepinephrine-reuptake inhibitors (SNRIs) | Venlafaxine |
|  | Milnacipran |
|  | Duloxetine |
| Pharmacokinetic DDI | |
| P-glycoprotein inhibitors and/or CYP3A4 inhibitors | Amiodarone |
|  | Clarithromycin |
|  | Cobicistat |
|  | Cyclosporine |
|  | Dronedarone |
|  | Itraconazole |
|  | Ritonavir |
|  | Azithromycin |
|  | Verapamil |
|  | Fluconazole |
|  | Voriconazole |
|  | Posaconazole |
|  | Imatinib |
| P-glycoprotein inducers and/or CYP3A4 inducers | Carbamazepine |
|  | Phenobarbital |
|  | Phenytoin |
|  | Rifampin |
|  | Enzalutamide |

**Supplement Table 2. Definition of comorbidities and clinical outcomes**

| Diagnosis | ICD-10-CM code | Diagnostic definition |
| --- | --- | --- |
| Atrial fibrillation | I480-484, I489 | Admission or outpatient department≥1 |
| Valvular atrial fibrillation | I050, I052, I059, Z952-Z954 |  |
| Pulmonary embolism | I26 | Admission≥1 |
| Deep vein thrombosis | I802 | Admission≥1 |
| Received joint replacement operation | N0711, N1711, N1721, N2070, N3710, N3721, N3717, N3720, N2072, N2077, N3722, N3727 | Admission≥1 |
| End stage renal disease | N185, Z49 | Dialysis≥2 |
| Ischemic stroke | I63, I64 | Admission≥1 and brain imaging (CT or MRI) ≥1 |
| Intracranial hemorrhage | I60-62 | Admission≥1 or RBC transfusion≥1 |
| Hospitalization for gastrointestinal bleeding | K22.6, K25.0, K25.2, K25.4, K25.6, K26.0, K26.2, K26.4, K26.6, K27.0, K27.2, K27.4, K27.6, K28.0, K28.2, K28.4, K28.6, K29.0, K62.5, K92.0, K92.1, K92.2 | Admission≥1 and RBC transfusion≥1 |
| Hospitalization for major bleeding | Intracranial bleeding or gastrointestinal bleeding | ICH, admission≥1 or RBC transfusion≥1 |
|  |  | GI bleeding, admission≥1 and RBC transfusion≥1 |
| Congestive heart failure | I50 | Admission or outpatient department≥1 |
| Hypertension | I10-I13, I15 | Admission≥1 or outpatient department≥2 |
| Diabetes mellitus | E11-E14 | Admission≥1 or outpatient department≥2 |
| Vascular disease |  |  |
| Prior myocardial infarction | I21, I22 | Admission or outpatient department≥1 |
| Peripheral arterial disease | I70, I73 | Admission or outpatient department≥2 |
| Systemic arterial thromboembolism | I74 | Admission≥1 |
| Transient ischemic attack | G459 | Admission≥1 |

**Supplement Table 3. DDIs related to ischemic stroke events**

|  | **Ischemic stroke**  **(N = 21)** | |
| --- | --- | --- |
|  | N | % |
| **Aspirin** | 15 | 71.4 |
| **Clopidogrel** | 5 | 23.8 |
| **Escitalopram** | 3 | 14.3 |
| **Ketorolac** | 2 | 9.5 |
| **Naproxen** | 1 | 4.8 |
| **Paroxetine** | 1 | 4.8 |
| **Amiodarone** | 1 | 4.8 |
| **Verapamil** | 1 | 4.8 |

**Supplement Table 4. DDIs related to hospitalization for major bleeding events**

|  | **Hospitalization for major bleeding**  **(N = 11)** | |
| --- | --- | --- |
|  | N | % |
| **Escitalopram** | 3 | 27.3 |
| **Aspirin** | 2 | 18.2 |
| **Clopidogrel** | 2 | 18.2 |
| **Aceclofenac** | 1 | 9.1 |
| **Ketorolac** | 1 | 9.1 |
| **Ketoprofen** | 1 | 9.1 |
| **Amiodarone** | 1 | 9.1 |
| **Azithromycin** | 1 | 9.1 |

**Supplement Table 5. Comparisons between patients with and without ICH**

|  | **ICH**  **(N = 11)** | | **No ICH**  **(N = 1,927)** | | **Odds ratio (95%CI)** | ***P*-value** |
| --- | --- | --- | --- | --- | --- | --- |
|  | N | % | N | % |  |  |
| **Age, median (IQR)** | 78 (76-82) | | 71 (62-78) | | 1.10 (1.03-1.19) | 0.009 |
| **Sex** |  |  |  |  |  |  |
| **Male** | 8 | 72.7 | 1,190 | 61.8 | REF | |
| **Female** | 3 | 27.3 | 737 | 38.2 | 0.61 (0.13-2.10) | 0.460 |
| **CHA2DS2-VASc** |  |  |  |  |  |  |
| **0-1** | 1 | 9.1 | 545 | 28.3 | REF | |
| **2-3** | 4 | 36.4 | 953 | 49.5 | 2.29 (0.34-44.81) | 0.460 |
| **≥ 4** | 6 | 54.5 | 429 | 22.2 | 7.62 (1.30-144.24) | 0.061 |
| **DOAC** |  |  |  |  |  |  |
| **Rivaroxaban** | 3 | 27.3 | 566 | 29.4 | REF | |
| **Apixaban** | 2 | 18.2 | 431 | 22.4 | 0.88 (0.12-5.31) | 0.884 |
| **Dabigatran** | 1 | 9.1 | 294 | 15.2 | 0.64 (0.03-5.04) | 0.701 |
| **Edoxaban** | 0 | 0 | 159 | 8.2 | 0 | 0.991 |
| **Mixed** | 5 | 45.4 | 477 | 24.8 | 1.98 (0.48-9.68) | 0.352 |
| **DDI** |  |  |  |  |  |  |
| **No** | 6 | 54.6 | 1,420 | 73.7 | REF | |
| **Yes** | 5 | 45.4 | 507 | 26.3 | 2.33 (0.67-7.78) | 0.163 |
| **Number of DDI** |  |  |  |  |  |  |
| **0** | 6 | 54.6 | 1,420 | 73.7 | REF | |
| **1** | 3 | 27.2 | 400 | 20.8 | 1.78 (0.37-6.76) | 0.419 |
| **≥ 2** | 2 | 18.2 | 107 | 5.5 | 4.42 (0.64-19.47) | 0.071 |

Intracranial hemorrhage, ICH

**Supplement Table 6. Comparisons between patients with and without hospitalization for GI bleeding**

|  | **GI bleeding**  **(N = 11)** | | **No GI bleeding**  **(N = 1,927)** | | **Odds ratio (95%CI)** | ***P*-value** |
| --- | --- | --- | --- | --- | --- | --- |
|  | N | % | N | % |  |  |
| **Age, median (IQR)** | 81 (76-88) | | 71 (62-78) | | 1.15 (1.06-1.25) | 0.001 |
| **Sex** |  |  |  |  |  |  |
| **Male** | 5 | 45.5 | 1,193 | 61.9 | REF | |
| **Female** | 6 | 54.5 | 734 | 38.1 | 1.95 (0.59-6.79) | 0.271 |
| **CHA2DS2-VASc** |  |  |  |  |  |  |
| **0-1** | 0 | 0 | 546 | 28.3 | 0 | 0.989 |
| **2-3** | 8 | 72.7 | 949 | 49.3 | 1.21 (0.35-5.56) | 0.775 |
| **≥ 4** | 3 | 27.3 | 432 | 22.4 | REF | |
| **DOAC** |  |  |  |  |  |  |
| **Rivaroxaban** | 2 | 18.2 | 567 | 29.4 | REF | |
| **Apixaban** | 5 | 45.4 | 428 | 22.2 | 3.31 (0.71-2.32) | 0.154 |
| **Dabigatran** | 0 | 0 | 295 | 15.3 | 0 | 0.993 |
| **Edoxaban** | 0 | 0 | 159 | 8.3 | 0 | 0.995 |
| **Mixed** | 4 | 36.4 | 487 | 24.8 | 2.37 (0.46-1.72) | 0.320 |
| **DDI** |  |  |  |  |  |  |
| **No** | 5 | 45.5 | 1,419 | 73.6 | REF | |
| **Yes** | 6 | 54.5 | 508 | 26.4 | 3.35 (1.01-11.68) | 0.047 |
| **Number of DDI** |  |  |  |  |  |  |
| **0** | 5 | 45.5 | 1.419 | 73.6 | REF | |
| **1** | 5 | 45.5 | 399 | 20.7 | 3.56 (0.99-12.85) | 0.046 |
| **≥ 2** | 1 | 9.1 | 109 | 5.7 | 2.60 (0.14-16.33) | 0.384 |

**Supplementary Figure 1. Study Population Enrollment Flow**


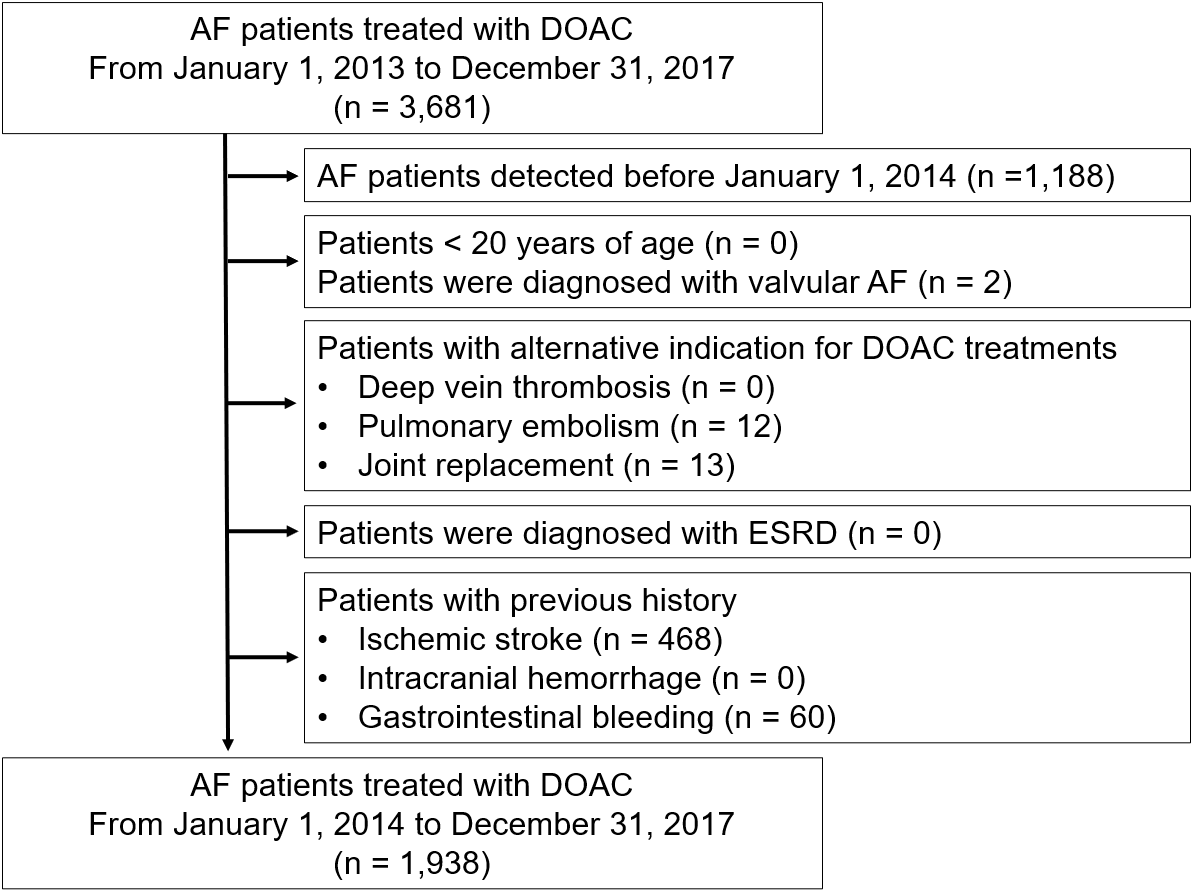

Supplement: Supplementary file 1 — Supplementary Information. [file 41598_2021_1786_MOESM1_ESM.docx]
